# Developing a process-oriented classroom observation protocol for assessing high school students’ computational thinking in science classrooms: a Rasch-based proficiency framework

**DOI:** 10.3389/fpsyg.2026.1722422

**Published:** 2026-02-20

**Authors:** Hui Zhao, Jian Yu, Gaofeng Li

**Affiliations:** 1 Faculty of Education, Shaanxi Normal University, Xi'an, China; 2 College of Teacher Education, Hebei Normal University, Shijiazhuang, China; 3 College of Life Sciences, Shaanxi Normal University, Xi’an, China

**Keywords:** computational thinking, high school, process-oriented classroom observation protocol, proficiency framework, Rasch analysis

## Abstract

**Introduction:**

Development of computational thinking (CT) in science education requires assessment tools that capture dynamic skill progression within authentic classroom settings. Existing assessment tools focus on CT overall performance with approximate sub-dimensions and level divisions, making it challenging to provide continuous, targeted guidance for fostering high school students’ CT competencies. This study aims to develop and validate the High School Students’ Computational Thinking Observation Protocol (HS-CTOP), a process-oriented tool for tracking high school students’ CT progression in science classrooms.

**Methods:**

Grounded in the Abstraction, Decomposition, Evaluation, Generalization, and Algorithmic thinking (ADEGA) framework, the HS-CTOP operationalizes CT into seventeen sub-dimensions and four proficiency levels. Data were derived from video-coded behaviors of 613 students across 120 biology classes in seven regions of mainland China, with validation conducted via the Rasch partial credit model.

**Results:**

The results confirm the HS-CTOP’s robust psychometric properties, including an item reliability of 0.96, person reliability of 0.83, and satisfactory unidimensionality. The protocol effectively distinguishes CT levels across different student groups.

**Discussion:**

The HS-CTOP provides educators with detailed insights into CT development patterns to inform differentiated instruction in science classrooms.

## 1 Introduction

Amid rapid technological advances of rapid technological advancement, computational thinking (CT) has emerged as a foundational competency transcending diverse social domains. As emphasized in global educational reforms (Lee et al., 2023; Luo et al., 2019; Wing, 2006), CT constitutes a core 21st-century skill, equipping individuals to tackle complex problems through algorithmic reasoning and systematic analysis. While CT originated in computational science—initially advocating for “thinking like computer scientists”—its broader significance lies in its capacity to deepen understanding across science and mathematics (Martins et al., 2023; Weintrop et al., 2016; Yadav et al., 2018). For example, integrating CT with science education enhances students’ grasp of scientific knowledge, learning outcomes, and practical skills (Lei et al., 2020). Within biology, specifically, CT is critical for both researchers and students to navigate the complexity of life processes. For instance, evolution operates across multiple temporal and organizational scales, requiring the systematic analysis and pattern recognition that CT enables (Guo et al., 2016).

Despite its growing significance, assessing CT remains a challenge. The current methods of paper-and-pencil tests, portfolios, surveys, and interviews focus on learning outcomes rather than the cognitive processes involved in CT (Tsai et al., 2021). They fail to provide process-oriented assessments functional for teaching CT and associated processes. For instance, Román-González et al. (2017) assessed CT by a test of multiple-choice questions measuring students’ computational concepts, the Computational Thinking Scale (CTS) assesses CT through 29 self-reported items (Korkmaz et al., 2017). Therefore, if science education aims to enhance student learning, CT assessment should directly target student actions, engagement, and learning within the classroom setting. Current research on CT assessment focuses on research scenarios rather than teaching scenarios (Tang et al., 2020). For educators, a practical research direction is to explore the applicability of these CT assessment tools in the classroom environment. Regarding the grade level, previous studies have primarily concentrated on elementary and middle schools (Han, 2023; Tang et al., 2020), which is not conducive to the assessment and development of CT among high school students.

While CT is integral to biology learning, conventional outcome-based assessment methods (e.g., paper-and-pencil tests) only measure final performance, failing to capture the dynamic cognitive processes underlying CT development (Tsai et al., 2021). This gap hinders formative feedback for teachers to adjust instruction. In contrast, video-based classroom observation enables non-intrusive, systematic documentation of students’ in-situ behaviors (e.g., experimental design discussions, data analysis reasoning), providing direct evidence of CT in action (Alonzo and Kim, 2016; Nieuwenhuis, 2014). However, existing CT observation protocols lack fine-grained sub-dimensions proficiency levels tailored to high school science contexts, limiting their utility for instructional decision-making.

To address these gaps, this study develops and validates the High School Students’ Computational Thinking Observation Protocol (HS-CTOP) in science classrooms, a novel instrument designed to assess high school students’ CT proficiency in science classrooms and capture their dynamics, thereby providing educators with refined teaching tracking and feedback. Unlike existing CT assessment tools that lack fine-grained proficiency hierarchies across sub-dimensions, the HS-CTOP operationalizes CT into 17 sub-dimensions within the ADEGA framework and establishes four empirically validated proficiency levels via the Rasch partial credit model—bridging the gap in process-oriented assessment tailored specifically to high school science teaching contexts. The integration of these elements enables the precise diagnosis of students’ dynamic CT development, rather than merely providing aggregate scores, and offers teachers actionable insights for differentiated instruction and curriculum refinement.

## 2 Literature review

### 2.1 Components of CT

CT continues to be a prominent topic of academic discussion, encompassing specialized programming skills to broad cognitive strategies (Kang et al., 2023; Zhang et al., 2024). In fact, CT is based on programming and computational concepts. For instance, Brennan and Resnick (2012) proposed a theoretical framework comprising three key components of CT: computational concepts, practices, and perspectives. Conversely, Weintrop et al. (2016) proposed a broader STEM (science, technology, engineering, and mathematics) integration, categorizing CT into data practice, modeling and simulation, computational problem-solving, and systems thinking. The International Society for Technology in Education (ISTE) defined CT as reflecting creativity, algorithmic thinking, critical thinking, problem-solving, cooperative thinking, and communication skills. Based on an experiment evaluating the impact of CT modules on preservice teachers, Yadav et al. (2014) outlined five aspects of CT: problem identification and decomposition, abstraction, logical thinking, algorithms, and debugging. These multiple definitions emphasize CT’s dual role: (1) a specialized tool in computer science and (2) a broad cognitive approach in STEM fields.

Notwithstanding the variation in CT definitions, common consensus (e.g., Hsu et al., 2018; Kalelioglu et al., 2016) shows that the core components of CT are closely related to cognitive skills, such as abstraction and algorithmic thinking for problem-solving. For instance, using a meta-analysis of multiple studies on CT, Selby and Woollard (2013) provided an operational definition of the components of CT: Abstraction, Decomposition, Evaluation, Generalization, and Algorithmic thinking (ADEGA). Abstraction (Ab) is the ability to decide between the key details of a problem and those that can be disregarded. Decomposition (De) breaks down complex problems into specific ones. Evaluation (Ev) ensures that an algorithmic solution fits a particular purpose. Generalization (Ge) is the ability to solve a problem in generic terms and produce solutions that apply to problems and share some characteristics with the original problem. Algorithmic thinking (Al) is a problem-solving approach that involves developing a series of sequential steps to achieve the expected results. These five competencies employ terminology from computer science, which aligns with the original definition of CT while overcoming disciplinary limitations. Furthermore, these elements are consistent with the general concept of problem-solving and play a crucial role in understanding, analyzing, and solving problems. Compared with Brennan and Resnick's (2012) framework which emphasizes programming-related concepts, the ADEGA framework is more suitable for science classroom observation because it focuses on core cognitive processes (eg, Ab, De) that align with the problem-solving logic in high school biology, such as decomposing the ‘photosynthesis rate experiment’ into variable control and data analysis steps.

The ADEGA framework has a profound intrinsic connection with CT in science, as its core dimensions align precisely with the cognitive logic and practices of scientific inquiry. Ab corresponds to scientific data practices—including multi-source data collection, pattern recognition, and system modeling—serving as the core for extracting key information from complex phenomena. De supports scientific problem-solving by splitting complex topics (e.g., ecosystem research) into actionable subtasks via component analysis and integrative decisions. Ev permeates scientific inquiry, ensuring the rationality of experiments and models through resource optimization, solution assessment, and trade-off analysis. Ge enables the transfer of scientific knowledge, from identifying cross-problem patterns to adapting solutions across disciplines, meeting the need to explore universal laws. Al provides structural support for scientific processes, regulating experiments and data processing through sequential step design, parallel operations, and procedural automation.

### 2.2 Assessments of CT

#### 2.2.1 Greater focus on outcomes rather than the process

Current assessments focus on the outcome of CT, neglecting the development process necessary to provide feedback for CT teaching. Portfolio assessment and observations are process-oriented assessment tools, providing students with beneficial feedback for further learning. Portfolio assessment enables students to comprehensively sort out the skills acquired through project practice or work results, making them uniquely valuable in the student evaluation system (Lui et al., 2020). However, these methods can only be implemented on specific programming or computing platforms (Chen et al., 2017). Next, multiple-choice and open-ended questions are typically based on the correctness and completeness of the answers, and such assessment primarily serve the purpose of summative evaluation. However, when students participate in practical projects, these assessment methods are complex to accurately reflect the process characteristics of CT learning (Fields et al., 2019). Interview and survey methods make it challenging to obtain deep-thinking information or empirical evidence from students in CT (Tang et al., 2020).

#### 2.2.2 From aggregate scores to a proficiency framework

Most assessment tools focus on the basic components of CT and do not delve into the sub-dimensions within each category. These tools reveal the comprehensive scores of students in all components, which makes identifying the source of CT deficiencies challenging. For instance, the CTS is a five-point Likert-type scale comprising 22 items covering five factors (Korkmaz et al., 2017); however, its scope is confined to certain specific subskills outlined in the definition provided by International Society for Technology in Education (2015). The CTt involves 14 sub-dimensions under three key components (Román-González et al., 2017). When considered within the CT framework (Brennan and Resnick, 2012), it emphasizes “computational concepts,” partially addresses “computational practices,” and overlooks “computational perspectives.”

When evaluating complex skills such as CT, their ability structure and hierarchical system must be considered (Seufert et al., 2021). Results indicating that “the student ranks within the top 10% of all test takers” or “the student answered 67% of the questions correctly” do not promote student learning. The proficiency model can achieve an enhanced interpretation of test results by utilizing projects with clear features that students can systematically master (American Educational Research Association, American Psychological Association, and National Council on Measurement in Education, 2014). Although people recognize the importance of building proficiency models for a deeper understanding of CT (Fraillon et al., 2019), research on the proficiency levels of CT remains nascent (Guggemos et al., 2023).

#### 2.2.3 Overlooked concern for high school science classrooms

The existing CT assessment tools are designed for primary and middle school students rather than high school students (Lee et al., 2022). Most of these assessment tools focus on curricula such as STEM, and further research is necessary to assess CT within science domains (Tang et al., 2020). Given that CT can enhance students’ comprehension of multidisciplinary knowledge, including STEM and non-STEM subjects, and help them solve daily problems (International Society for Technology in Education and Computer Science Teachers Association, 2011; Weintrop et al., 2016), additional assessments must be developed to emphasize the synergistic effects between CT and domain-specific knowledge. Without being fully integrated into the school curriculum system, assessment of CT development among teachers, students, and teaching plans will be imprecise (Cutumisu et al., 2019).

#### 2.2.4 Psychometric properties of assessment tools require enhancement

Validity and reliability ensure that the CT assessment provides reliable information on student skills. However, the psychological characteristics of existing CT assessment tools must be further strengthened (Cutumisu et al., 2019; Yusoff et al., 2020). For instance, the Computational Thinking Test (CTt) (Román-González et al., 2017) has been criticized for its limited coverage of CT sub-dimensions, which may compromise its construct validity. Due to the ambiguous definition of psychological constructs in current assessment instruments, inconsistencies frequently occur in outcomes, complicating the comparison of studies and the generalization of their findings across a wide array of educational contexts (Chan et al., 2021).

#### 2.2.5 Classroom observation for CT assessment advantages values and key limitations

Unlike surveys (which rely on self-reported data) or portfolios (which focus on final products), classroom observation captures student behaviors (e.g., experimental design discussions) that reflect the dynamic cognitive process of CT, making it more suitable for tracking CT progression in science classrooms (Alonzo and Kim, 2016). Nieuwenhuis (2014) argues that observation centers on the systematic collection of information through non-intrusive visual means, thereby avoiding direct interaction with participants. Researchers can generate valid and reliable scores for predefined assessment indicators in educational settings by systematically reviewing classroom video data and applying a validated observation protocol (He et al., 2016). The ratings assigned by observers using all indicators in the assessment tool can be compiled and analyzed with other measurement methods (Liu, 2012). Compared to on-site observation, classroom videos reflect the inherent “complexity and subtlety” of teaching - researchers can use the slow playback or repeated review characteristics of videos to comprehensively capture the process of classroom activities and analyze the teaching process from multiple perspectives (Alonzo and Kim, 2016; Xu and Clarke, 2018). Focusing on teaching processes, classroom observation provides effective feedback—a critical factor for fostering students’ CT. This approach captures the dynamic nature of CT and supports the iterative refinement of teaching strategies, enhancing students’ learning outcomes.

However, like existing assessment tools, the lack of unified and reliable observational protocols and standards for CT remains a core issue in current research. Existing observational protocols predominantly rely on researcher-developed checklists or non-standardized frameworks, resulting in substantial discrepancies in observation dimensions and coding rules across studies (Clarke-Midura et al., 2023; Ocampo et al., 2024). Moreover, most of these tools lack systematic psychometric validation, with insufficient reliability and validity evidence, which severely undermines the comparability and credibility of research findings. Second, the absence of standardized observational protocols compromises the reproducibility of results. Current studies lack unified guidelines for key parameters such as observation duration, recording methods (e.g., field notes, video coding), and data processing rules (McCormick and Hall, 2022; Monteiro et al., 2021). This makes it challenging to replicate and verify findings across different contexts, thereby weakening the generalizability of conclusions. Furthermore, the majority of existing CT observational protocols are designed for computational or programming-specific environments and cannot be directly adapted to science classroom contexts (Kalliopi and Michail, 2019; Gillott et al., 2020), especially in high school. This misalignment further constrains their utility in broader educational scenarios, restricting the comprehensive assessment of CT in integrated learning environments.

In summary, there is currently a lack of professional CT observation protocols with high reliability and validity that focus on finer-grained CT sub-dimensions and are specifically designed to evaluate students’ process-oriented performance in CT learning within high school science classrooms. This gap significantly undermines the feedback and guiding value of observation results for CT instruction, making it challenging for frontline teachers and researchers to capture the dynamics of students’ CT development in authentic learning contexts. Therefore, developing such rigorously validated and fine-grained professional CT observation protocols is an urgent priority.

## 3 Research questions

To develop the High School Computational Thinking Observation Protocol (HS-CTOP), a tool with robust psychometric properties specifically designed to assess students’ CT performance in high school science classrooms, this study aims to enable more nuanced tracking and feedback on students’ CT development within the context of science instruction. Specially, this study proposes the following three research questions:

RQ1: What is the construct validity and internal reliability of HS-CTOP in assessing CT development among high school students?

RQ2: Do the hypothesized four proficiency levels of CT in the HS-CTOP align with the empirical data from high school biology classrooms?

RQ3: Can the performance indicators and levels of the HS-CTOP effectively distinguish CT proficiency among high school students across different grades and regions?

Each research question is supported by targeted evidence from Rasch model analyses and empirical verification. To elaborate, RQ1 on the HS-CTOP’s construct validity and internal reliability will be addressed via unidimensionality tests, item/person reliability coefficients, and item fit statistics; RQ2 on the alignment of the hypothesized four CT proficiency levels with empirical biology classroom data will be verified through category functioning analysis and level-threshold mapping; RQ3 on the HS-CTOP’s discriminative ability across student grades and regions will be demonstrated via CT proficiency group comparisons and performance level distribution analysis.

## 4 Methods

### 4.1 Development of theoretical framework

#### 4.1.1 Sub-dimensions of ADEGA and operational definition

**Figure 1:**
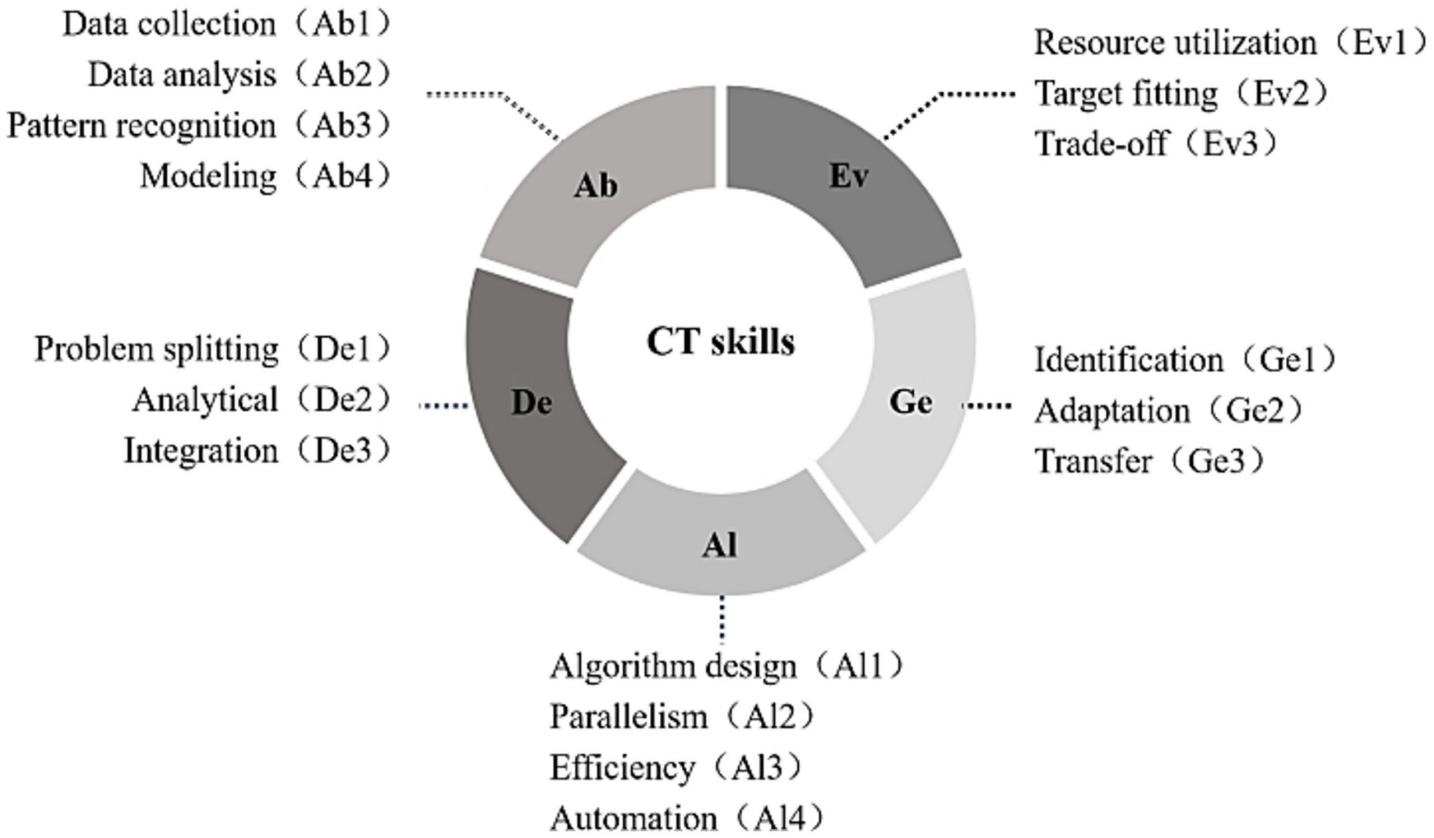
Sub-dimensions of computational thinking.

Based on previous studies (e.g., Csizmadia et al., 2015; Shute et al., 2017; Tsai et al., 2021), 17 sub-dimensions of CT are identified under the ADEGA framework (Figure 1).

Abstraction (Ab) comprises four sub-dimensions: data collection (Ab1), collecting the relevant and important information from multiple sources; data analysis (Ab2), understanding the relationships among multilayered datasets; pattern recognition (Ab3), identifying patterns/rules underlying the data/information structure; and modeling (Ab4), building models or simulations to represent how a system operates, and/or how a system will function in the future. Decomposition (De) includes three sub-dimensions: problem splitting (De1), breaking down tasks; analytical thinking (De2), thinking about problems in terms of component parts; and integration (De3), making decisions about dividing into subtasks with integration in mind, for example, deduction. Algorithm (Al) consists of four sub-dimensions: algorithm design (Al1), creating a series of ordered steps to solve a problem; parallelism (Al2), carrying out a certain number of steps simultaneously; efficiency (Al3), designing the fewest number of steps to solve a problem, removing redundant and unnecessary steps; and automation (Al4), automating the execution of the procedure when required to solve similar problems. Evaluation (Ev), encompasses three sub-dimensions: resource utilization (Ev1), making decisions about the good use of resources; target fitting (Ev2), assessing whether an artifact is fit for a given purpose; and trade-off (Ev3), thinking of the best solution for a program. Finally, generalization (Ge) involves three sub-dimensions: identification (Ge1), identifying patterns and commonalities in problems, processes, solutions, or data; adaptation (Ge2), adapting solutions or parts of solutions so that they apply to a whole class of similar problems; and transfer (Ge3), transferring ideas and solutions from one problem area to another.

Despite the adoption of this framework in several studies (e.g., Çoban and Korkmaz, 2021; Peters-Burton et al., 2023), its development into a reliable assessment tool remains insufficient, a practical assessment of these skills needs to be addressed.

#### 4.1.2 Assumptions of proficiency levels

Given the limited duration of individual class sessions, high school students face challenges in completing all CT-related processes or activities within a single lesson. We posit that students capable of fulfilling complex CT-related observational indicators or tasks can also complete less demanding ones. Therefore, each observation indicator needs to be categorized according to its difficulty level—even indicators within the same category may exhibit differences in difficulty. For instance, Handayani et al. (2022) found that students’ abstraction skills are evident during data collection and analysis, particularly when identifying patterns in data; by contrast, students’ abstraction skills appear less developed when creating models or simulations. Consistent with these findings, the complexity of Ab4 and Ab3 is anticipated to be greater than Ab2 and Ab1.

Building on the ADEGA framework, its associated indicators, and the difficulty order outlined in relevant literature (e.g., Handayani et al., 2022; Peters-Burton et al., 2023; Sulsilah et al., 2023) and theory. For instance, drawing on cognitive load theory (Sweller, 1988), we explain that students tend to master basic efficiency optimization (Al3) and parallel operation (Al2) before constructing complex sequential algorithms (Al1), as the latter requires integrating multiple steps into a coherent logical framework with higher cognitive demands. And this ordering aligns with the CT skill development trajectory identified by Peters-Burton et al. (2023), who demonstrated that automation (Al4) emerges as a high-order skill only when students can flexibly transfer algorithm design to similar problems. Finally, we hypothesize CT performance levels for high school students in science classrooms (Table 1).

**Table 1:** Hypothetical CT performance levels and students’ corresponding characteristics.

Components	Simple			Complex
	Level 1	Level 2	Level 3	Level 4
(Ab) Abstraction	Collect relevant information from multiple sources (Ab1)	Understand relationships among multilayered datasets (Ab2)	Identify patterns/rules underlying the data/information structure (Ab3)	Build models or simulations to represent how a system is designed to function in the future (Ab4)
(Al) Algorithmic thinking	Design the fewest number of steps to solve a problem, removing redundant steps (Al3)	Perform certain number of steps simultaneously (Al2)	Create a series of sequential steps to solve a problem (Al1)	Automate execution of the procedure when required to solve similar problems (Al4)
(De) Decomposition	Break down tasks (De1)	Effectively make decisions about dividing tasks into subtasks while considering integration (e.g., deduction) (De3)	Think about problems in terms of parts (De2)	
(Ev) Evaluation	Make decisions about using resources effectively (Ev1)	Develop the best solution for a program (Ev3)	Assess if an artifact is suitable for the purpose (Ev2)	
(Ge) Generalization	Identify patterns and commonalities in problems, processes, solutions, or data (Ge1)	Adapt solutions or parts of solutions so that they are applicable to similar problems (Ge2)	Transfer ideas and solutions from one problem area to another (Ge3)	

The CT indicators and levels listed in Table 1 are theoretically constructed and need further validation through empirical research.

It should be noted that the difficulty levels of CT sub-dimensions presented in Table 1 reflect the typical cognitive development patterns in high school science classrooms, rather than a fixed hierarchical relationship. As a cognitive skill deeply intertwined with task contexts, the difficulty of specific CT sub-dimensions may dynamically change depending on the complexity of instructional tasks and the characteristics of subject content. For instance, in simple data recording tasks such as observing plant cell division and counting data, the cognitive demand of Ab3 (Pattern recognition) might be lower than that of Ab2 (Understanding relationships among multilayered datasets). However, in complex system modeling tasks such as constructing population change models, the difficulty of Ab3 (identifying patterns/rules underlying population growth data) can increase significantly, even surpassing that of Ab4 (Building preliminary models or simulations to represent system functions). Furthermore, students’ prior subject knowledge such as their mastery of biological concepts may also influence the perceived difficulty of sub-dimensions. For students proficient in genetic principles, the difficulty of Ge3 (Transfer) in genetic probability calculation tasks may be reduced. Therefore, the hierarchical classification in this study aims to provide a “baseline framework” for instructional assessment, which should be flexibly adjusted in practical applications based on specific instructional tasks such as experimental design and theoretical analysis and students’ subject knowledge foundations.

### 4.2 Sample

Biology classrooms were selected as the setting to test the psychometric properties of the HS-CTOP for several reasons. The inherent complexity of biology and the frequent application of CT in modeling biological systems, such as ecosystem dynamics and genetic analysis, closely align the discipline’s tasks with the core CT dimensions (e.g., Ab, De). A variety of high school biology modules (e.g., “Molecules & Cells,” “Homeostasis & Regulation”) can comprehensively cover the measurement scope, supporting multidimensional discriminability checks. The practical, experiment-centered nature of biology classrooms allows for the observation of dynamic CT processes, which aligns with the HS-CTOP’s process-oriented assessment focus and provides high-quality empirical data for verifying properties such as reliability and item fit.

To validate the psychometric properties of this tool, we employed maximum variation sampling, which is a form of purposive sampling, to strategically select samples that span diverse scenarios and meet the measurement requirements of the HS-CTOP. When developing classroom observation protocols, a sample size of more than 100 classes is generally deemed sufficient for assessing construct validity (Boomsma, 1985). To effectively validate the HS-CTOP and analyze the distribution of CT among high school science students, our sample included over 120 video-recorded biology lessons from seven regions across mainland China, covering five high school biology textbooks (Table 2). These regions were chosen to represent varied educational contexts nationwide, reflecting diverse socio-economic and educational landscapes to ensure ecological validity. Given that the new curriculum concludes in the second year of high school, the video-recorded lessons included both Grade 10 and Grade 11.

**Table 2:** Descriptive information of video-recorded biology lessons.

Lesson sources		Count	Percentage
Districts	Northwest	7	5.83
	Southwest	16	13.33
	Northeast	8	6.67
	North	7	5.83
	Central	23	19.17
	East	22	18.33
	South	37	30.83
Grades	Grade 10	74	61.67
	Grade 11	46	38.33
Textbooks	Compulsory 1	32	26.67
	Compulsory 2	25	20.83
	Selective compulsory 1	20	16.67
	Selective compulsory 2	22	18.33
	Selective compulsory 3	21	17.50

Different teaching modules emphasize distinct CT processes. For example, Compulsory 1 (“Molecules & Cells”) focuses on microscopic and abstract content, with experiments that engage CT processes such as Ab, Al, De, and Ev. In contrast, Selective Compulsory 1 (“Homeostasis & Regulation”) addresses complex, diverse life processes, involving CT skills like Ab, De, and Ge.

Video recordings were collected from regular classroom sessions, with biology teachers and students participating as volunteers. They were informed that all lessons and associated data would be anonymized and used exclusively for research on CT in classroom instruction. While the direct units of observation in this study are regular biology classrooms, the core subjects of assessment are the high school students within these classrooms. For each video-recorded class, 6–8 students are selected through simple random sampling (covering different seating areas) to ensure the representativeness of students. All Rasch partial credit model analyses were conducted at the individual student level, with a final sample size of *N* = 613 (6–8 students per class across 120 biology lessons).

### 4.3 Coding

The HS-CTOP is based on 17 hypothetical CT indicators in Figure 1. Each item /indicator is scored using a partial credit model (3 points, 2 points, 1 point, 0 points, and missing/no scores) (Boone and Staver, 2020), consistent with specific coding rubrics, where 0 = not observed, 1 = below expectation, 2 = up to expectation, and 3 = above expectation. These rubrics were developed based on relevant literature (e.g., Peters-Burton et al., 2023; Sulsilah et al., 2023), classroom observations, and interviews with high school biology teachers engaged in CT teaching. Examples of questions posed in these interviews include: Which core biological concepts do you usually choose to integrate for CT cultivation, and which CT sub-dimensions do students most often struggle with?

We conducted a pilot test for the HS-CTOP. Wright and Tennant (1996) suggested that if each participant responds to ten or more items, the target sample size of 50 participants can ensure a 99% confidence level for Rasch analysis, and the estimated difficulty of the items will fall within one logit of the actual values. Using the pilot study, we extracted 50 biology lessons (more than 50 students) from the database. When a student’s CT was shown multiple times, the score for a specific item/indicator was determined by the average score achieved. Effective indicators vary by video, depending on the educational content.

To ensure the reliability of the rating system, we recruited three raters with relevant expertise aligned with the assessment context: an expert teacher specialized in high school biology teaching with CT integration; a biological education scholar focused on science education assessment; and a CT researcher experienced in CT teaching. They completed a 10-h training program to standardize scoring procedures, which included rubric calibration, case discussions, and four structured modules: two theoretical lectures on CT and the HS-CTOP, each lasting 2 h; 4 h of coding practice using 5 sample videos; 3 h of group deliberations on ambiguous coding cases; and a 1-h post-training test featuring two additional videos. After the initial training (Fleiss’ kappa = 0.832), a 20% stratified random subset of the main dataset (24 lessons, 123 students) was double-coded independently by all three raters to assess operational reliability. The post-coding Fleiss’ kappa was 0.796, indicating substantial agreement (Landis and Koch, 1977). No significant rater drift was observed, as the agreement level was consistent with the training phase. For the remaining 80% of videos, raters coded different region-stratified subsets to avoid regional bias.

The initial version of the HS-CTOP was revised through pilot testing. For instance, the probability curve should show distinct peaks for each scoring category at specific proficiency levels, indicating clear performance differentiation. During initial testing, Level 3 lacked a distinct peak in Al1, indicating a limited ability range and measurement inadequacy. We improved the scoring rubric by integrating Level 3 to enhance discriminant validity and simplify the application. The ultimate HS-CTOP coding scheme is detailed in Supplementary material 1, with 17 items/indicators. An instance of the coding rubric for “Algorithmic thinking” (Al) is presented in Supplementary material 2.

The core unit of observation was defined as student-specific behavioral episodes during classroom activities (e.g., experimental design discussions, data analysis reports). For sub-dimensions unrelated to course content (e.g., ‘automation’ in theoretical memory-based courses), data were coded as ‘not observed (NO)’ and treated as negligible missing data in Rasch analysis (Linacre, 2013). Evidence gaps caused by video quality issues were excluded from student datasets to avoid systematic bias.

The coding scheme was subsequently applied to evaluate students’ performance across 120 videos, which included 613 students, with each video lasting 40 min. The pilot test involved three raters who conducted the coding process individually for each indicator, following the principle of “regional balance.” Each rater was assigned 40 videos, encompassing 2–3 of the 7 sample regions, to prevent region-specific biases from influencing the coding outcomes. This standardized approach ensured the consistency and objectivity of the coding standards, offering reliable data to support the validation of HS-CTOP.

### 4.4 Data analysis

To validate the instrument, we used the partial credit Rasch model (Wright and Masters, 1982) to guide the design and revision of the measurement instrument, which allows for the calculation of confidence intervals for “measured values” or parameter estimates (Bond and Fox, 2015; Boone et al., 2014). Winsteps 3.74.0 was used to conduct the Rasch analysis. Studies validating assessment tools through Rasch models provide methodological support for this study (He et al., 2016; Zhang and Browne, 2023).

As noted by Bond and Fox (2015), items and response data are analyzed while applying the Rasch model to assess how well an individual’s responses align with the measurement model. The Rasch model requires unidimensionality (Bond and Fox, 2015; Boone et al., 2014) to ensure that the HS-CTOP indicators closely relate to and accurately measure CT. Boone and Staver (2020) indicated that a principal component analysis of residuals can effectively test the unidimensionality hypothesis of measurement tools. A variance explained by the Rasch dimension of 50% or greater can be considered evidence that the scale is unidimensional (Linacre, 2013). If the strength of the second dimension (the first contract) is less than three items (in terms of eigenvalues) and the unexplained variance of the first comparison is less than 5% (Oon and Subramaniam, 2011), it is assumed that the scale is unidimensional. Point-measure correlation refers to the correlation between observed data values and the overall measurement values of the items (or persons) that generated the observed values (Linacre, 2013). The mean square residual (MNSQ) is used to assess the degree of fit and examine the coherence of each item with the Rasch model. A Wright map plots a person’s estimated ability and an item’s estimated difficulty on the same logit scale (Neumann et al., 2013), evidencing construct validity for the instrument. Additionally, the degree of fitness, item reliability, and person reliability were assessed in the Rasch analysis.

When employing the HS-CTOP to assess students’ CT, we assigned a Rasch measure to an observed student. To grade students’ progress levels based on Rasch scores, we use each level’s estimated average items/thresholds as the basis for dividing the cognitive CT level (Shi et al., 2021; Zhang and Browne, 2023).

## 5 Results

### 5.1 Validity of HS-CTOP indicators

Boone and Staver (2020) emphasized two critical validity components in Rasch analysis: (1) model-data fit validity (assessed through item/person fit statistics) and (2) construct validity (evaluated via Wright map and discriminant validity). For the HS-CTOP, the Rasch dimension explained 45.8% of the total variance, slightly below the commonly cited 50% threshold for unidimensionality (Linacre, 2013). However, this conclusion is still defensible based on complementary evidence: first, the first contrast accounted for only 4.7% of the unexplained variance, well below the 5% cutoff for cross-dimensional contamination (Oon and Subramaniam, 2011); second, eigenvalues for the first and second contrasts (1.9 and 1.6, respectively) were both below 2.0, indicating no distinct secondary dimensions.

To further examine the construct validity of the HS-CTOP, we analyzed inter-dimension correlations among the five CT components using ConQuest 3.0. The Pearson correlation coefficients ranged from 0.41 to 0.73 (all *p* < 0.01; Table 3), falling within the recommended interval of 0.3–0.8 by Kline (2015) for establishing dimensionality. The high correlation between Ge and Ab (r = 0.73) may be due to the fact that high school biology requires students to abstract patterns (e.g., genetic inheritance laws) first, then generalize them to new scenarios (e.g., predicting offspring traits). The Expected *A Posteriori* (EAP)/Plausible Values (PV) reliability coefficient was above 0.7. While these correlations indicate shared variance across components, the absence of coefficients exceeding 0.80 mitigates concerns about multicollinearity, supporting the instrument’s capacity to measure distinct yet interrelated facets of CT. Combined with the above residual variance analysis, these results reinforce the unidimensionality of the HS-CTOP while preserving meaningful differentiation between its theoretical sub-dimensions.

**Table 3:** Correlation coefficients among the five potential components, indicating that these five dimensions correlated with each other.

Dimension	Dimension					EAP/PV reliability
	Ab	Al	De	Ev	Ge	
Ab						0.83
Al	0.62^*^					0.92
De	0.55^*^	0.66^*^				0.85
Ev	0.68^*^	0.41^*^	0.64^*^			0.76
Ge	0.73^*^	0.52^*^	0.46^*^	0.65^*^		0.81

^*^p < 0.01.

Ab, abstraction; Al, algorithmic thinking; De, decomposition; Ev, evaluation; Ge, generalization.

The HS-CTOP demonstrated strong psychometric properties, with item reliability (0.96) and person reliability (0.83) exceeding the 0.70 threshold, indicating strong consistency in the central concepts of item difficulty and person ability measures (Bond and Fox, 2015). The item separation (5.00) exceeded the recommended threshold of 3.00, confirming effective discrimination between item difficulty levels. Similarly, the person separation index (2.94) exceeded 2.00 (Bond and Fox, 2015), indicating sufficient sample heterogeneity to reliably differentiate students’ abilities.

The fitting statistics of most items in the revised HS-CTOP indicate that the infit and outfit mean-square (MNSQ) values fall within the acceptable range of 0.7 to 1.3, representing satisfactory model-data fit. However, items Al4 and Ev3 show slight misfit (Infit MNSQ < 0.7), indicating an “overfitting” pattern where students’ response patterns are excessively consistent. This may result from the scoring rubric’s “insufficient discriminative power” or the “limitations of the task context” in high school biology classrooms. Specifically, Al4 requires students to “automate procedure execution for similar problems,” but scenarios of “repeatedly solving similar problems” are scarce, for example, the same experimental design is usually implemented only once, leading to limited variability in student performance. Ev3 demands students to “weigh the best solution,” yet classroom time constraints often allow only one or two solutions, making it hard to demonstrate the “trade-off process” and resulting in convergent responses. Furthermore, Al4 is a high-order core sub-dimension of Al, and Ev3 is a critical indicator of Ev; both are integral to the ADEGA framework, and their removal would compromise the structural integrity of the measured CT construct. Additionally, the psychometric analyses confirmed they do not undermine the scale’s unidimensionality. Balancing theoretical completeness with empirical validity, the research team opted to retain the items, with the expectation that more targeted instructional scenarios or improved scoring rubrics would increase response variability in subsequent implementations.

The majority of items showed strong positive point-measure correlations (≥0.5), indicating good alignment between item performance and overall CT ability. Six items (Ab1, Ab2, Al1, De1, Ev1, Ev2) had relatively low correlations (0.32–0.47), suggesting weaker discrimination for these sub-dimensions (Table 4). The low correlations of these items may stem from context-specific constraints: for instance, Ab1 had a correlation of 0.36, likely because high school biology classrooms rarely provide opportunities for students to collect multi-source data independently. Future revisions should design targeted classroom activities to enhance students’ multi-source data collection skills, thereby improving the discriminant validity of Ab1. A potential cause for the low Pt-Measure Corr. of Ev1 may lies in the unreasonable gradient design of its scoring levels and insufficient discriminant validity—for instance, the cognitive difficulty gap between Level 1 (partial estimation) and Level 2 (estimation with inaccuracies) is minimal, making it hard to clearly distinguish students’ performance and thus resulting in a clustering of students’ scores in the middle range. Such a “floor effect” or “ceiling effect” will reduce the item’s discriminative power, thereby compromising the Pt-Measure Corr.

**Table 4:** Fit statistics for the 17 items in the revised HS-CTOP.

Item	Measure (logit)	Infit MNSQ	Outfit MNSQ	S.E. (logit)	Pt-Measure Corr.
Ab1	2.99	1.09	1.01	0.14	0.36
Ab2	−2.64	1.08	1.15	0.14	0.49
Ab3	−2.21	1.03	1.03	0.12	0.56
Ab4	−0.34	0.86	0.82	0.14	0.78
Al1	0.42	1.23	1.32	0.18	0.44
Al2	−0.16	0.88	0.82	0.18	0.68
Al3	−0.60	0.86	0.83	0.26	0.66
Al4	0.66	0.66	0.63	0.24	0.76
De1	0.78	1.15	1.12	0.32	0.47
De2	1.81	0.85	0.87	0.39	0.60
De3	0.97	1.00	1.02	0.39	0.51
Ev1	0.45	1.10	1.11	0.34	0.45
Ev2	0.88	1.24	1.24	0.57	0.32
Ev3	0.56	0.69	0.63	0.68	0.68
Ge1	−2.53	0.99	0.99	0.21	0.66
Ge2	−0.91	0.87	0.85	0.21	0.82
Ge3	−0.12	0.87	0.79	0.23	0.81

**Figure 2:**
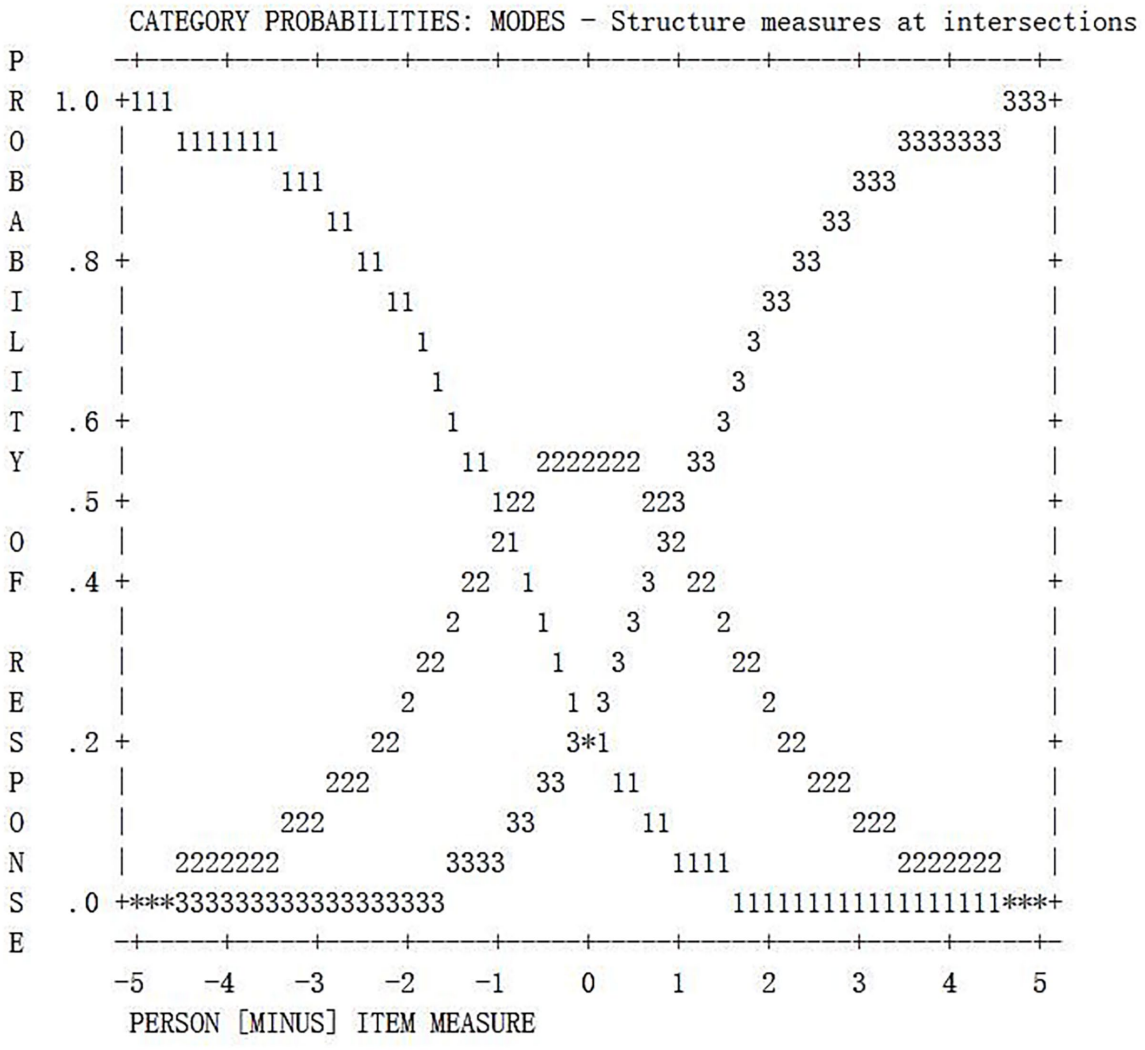
Probability curve for Ab1. Each rating category shows a significant peak, indicating the effectiveness of the Ab1 rating scale.

**Figure 3:**
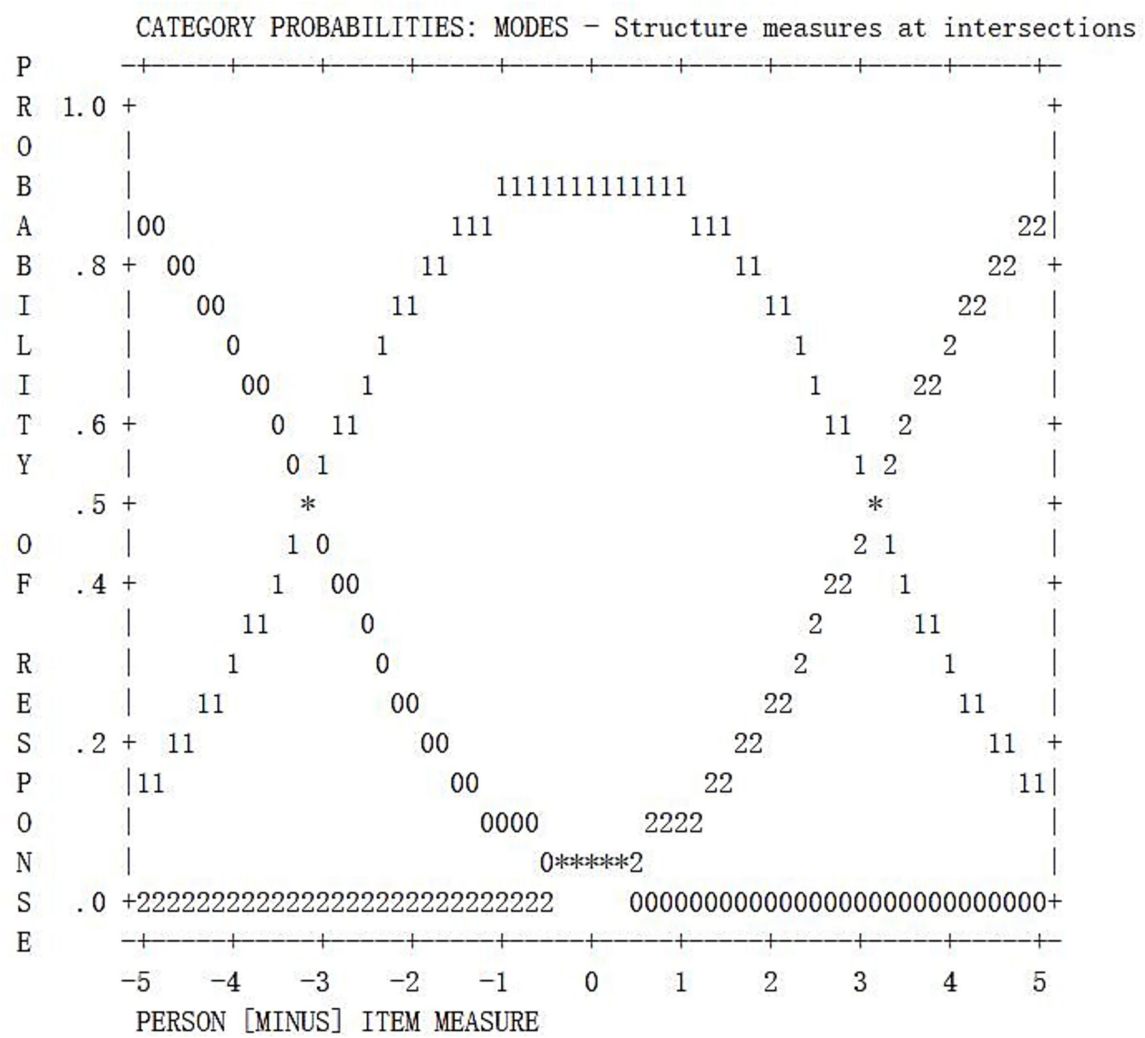
Probability curve for Ge1. Each rating category shows a significant peak, indicating the effectiveness of the Ge1 rating scale.

In addition, from the probability curves of each item, the corresponding category probability curves exhibit good discrete characteristics (e.g., the probability curve of Ab1 is shown in Figure 2, and Ge1 is shown in Figure 3). Furthermore, the category threshold estimation shows a monotonically increasing trend within the category. The interval between adjacent thresholds exceeds 1.1 logits, consistent with the guiding principle proposed by Linacre (2002). This suggests that the validity of the partial credit coding rubrics developed for each task in the study (Boone et al., 2014) is supported, as item scores demonstrate notable discriminant validity.

**Figure 4:**
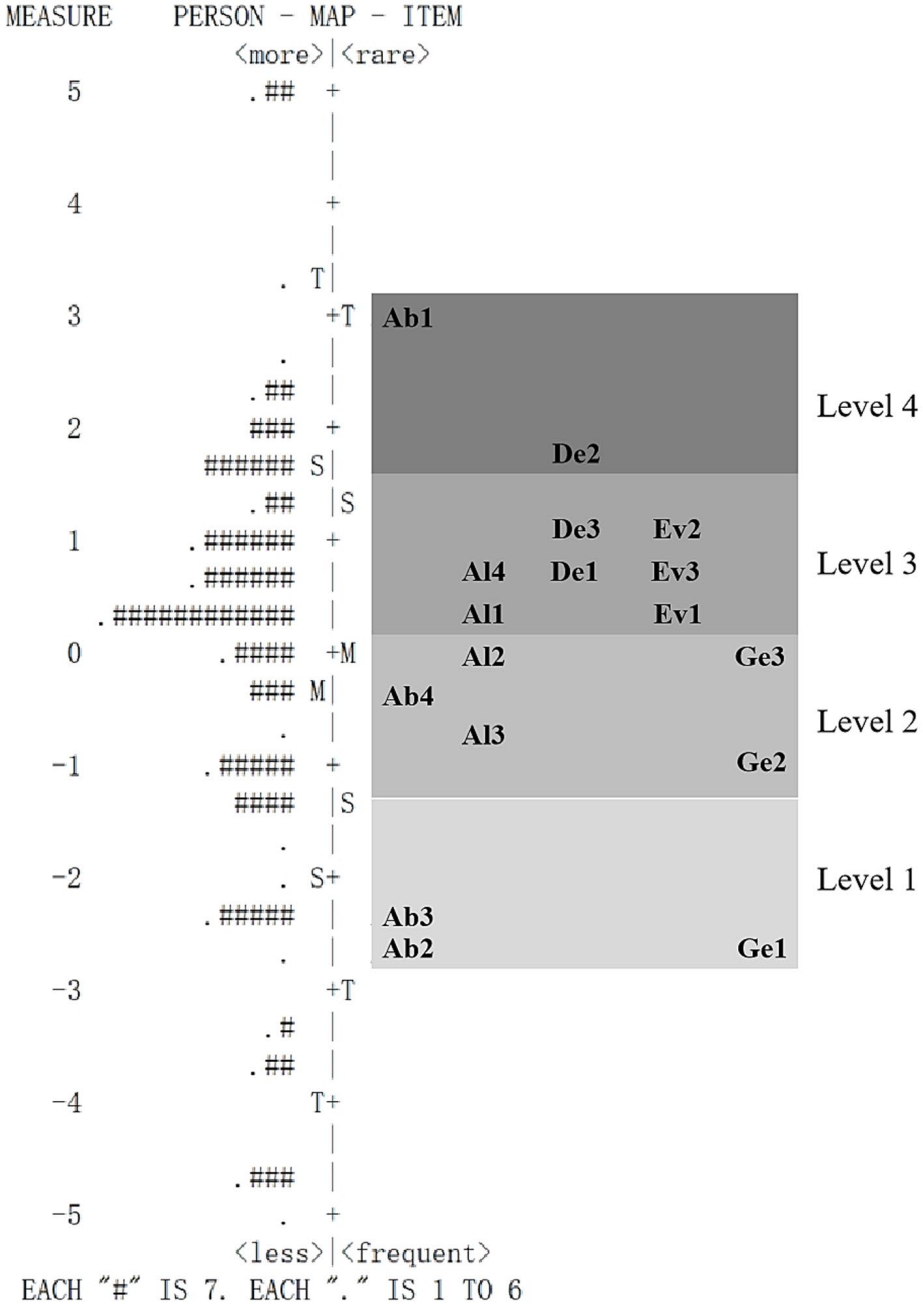
Wright map of the revised HS-CTOP.

**Figure 5:**
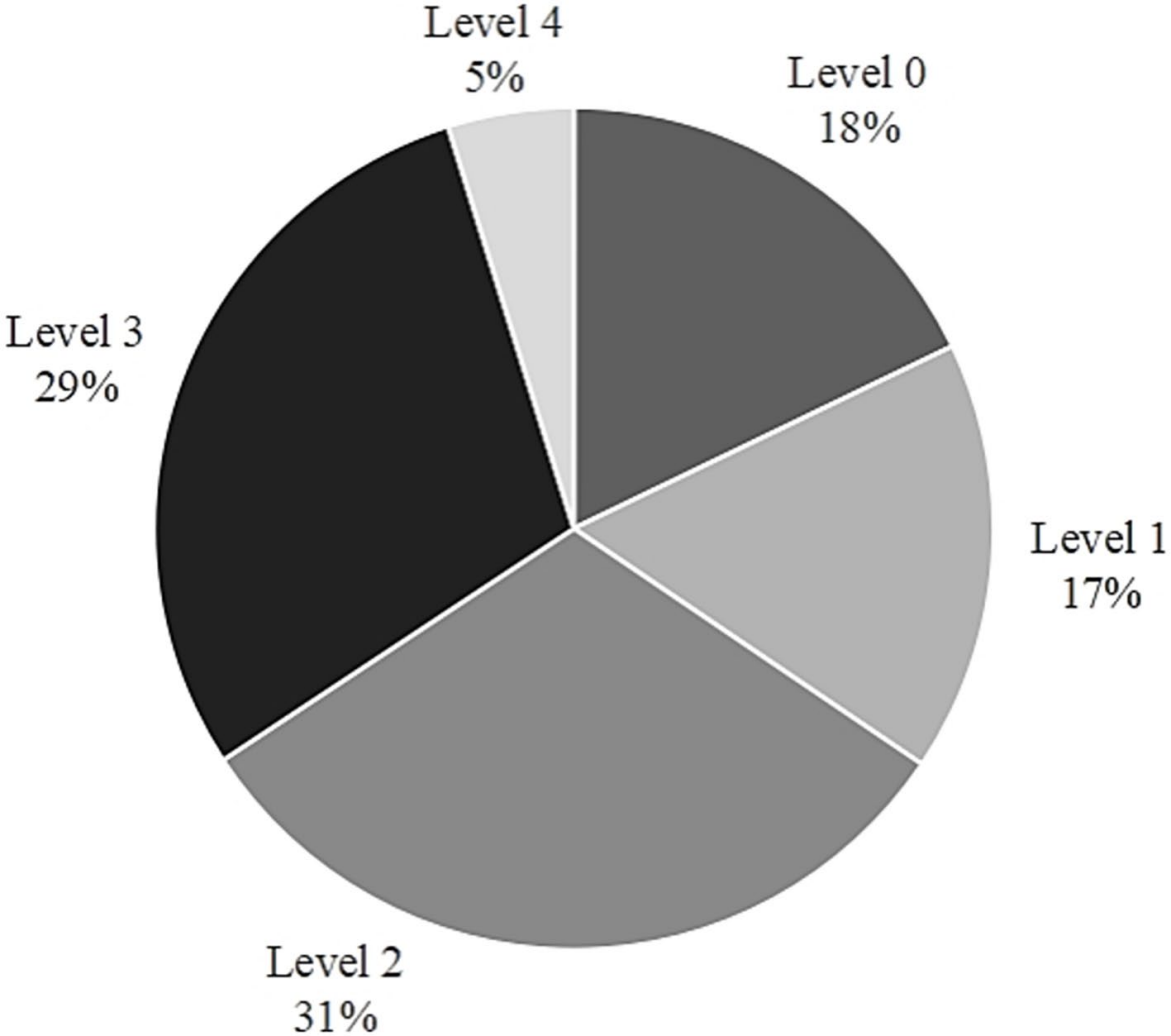
Distribution of CT performance by level.

In this study, students with superior CT are positioned higher on the Wright map. Items/indicators with higher difficulty measures are more challenging for students. The findings denote a substantial variation in the distribution of CT among students, ranging from −5.14 logits to 5.08 logits. The revised indicators’ difficulty levels varied, ranging from −2.64 logits to 2.99 logits (Figure 4). The Wright map also shows that the distribution of student abilities and item/indicator measurements has a good fit, indicating that the relevant indicators can effectively distinguish students with distinct levels of CT. Finally, the order of item difficulty corresponded with our expectations outlined in Table 1, except for Ab1 (Figure 5). The high difficulty of Ab1 may arise from the constraints of the conventional high school biology teaching model, which often depends on pre-prepared data sources for experiments. As the video illustrates, students seldom have the chance to independently gather information from various authentic sources, such as field observations and academic databases. Consequently, they lack training in “multi-source data screening and integration,” which is a core requirement of Ab1—to collect effective multi-source information. Due to the scarcity of practical opportunities, students exhibit significant deficiencies in this aspect.

The above-mentioned evidence covering components, reliability, item-fitting statistics, item-category probability, and Wright maps confirms the validity of the HS-CTOP.

Based on this comprehensive analysis, the HS-CTOP demonstrated robust validity (unidimensional structure, model-data fit) and reliability (high separation indices), consistent with the psychometric standards for classroom observation protocols.

### 5.2 HS-CTOP levels and characteristics

Based on a comparative analysis of the Rasch measurement measures/scores observed by students and different levels of CT, we divided students into groups according to their CT levels (Table 5).

**Table 5:** Measure ranges of different CT levels.

Item	Minimum (logit)	Maximum (logit)	Average (logit)	Range (logit)	Level
				(2.4, +∞)	Level 4
Ab1, De2	1.81	2.99	2.4	(0.67, 2.4)	Level 3
Al1, Al4, De1, De3, Ev1, Ev2, Ev3	0.42	0.97	0.67	(−0.43, 0.67)	Level 2
Ab4, Al2, Al3, Ge2, Ge3	−0.91	−0.12	−0.43	(−2.46, −0.43)	Level 1
Ab2, Ab3, Ge1	−2.64	−2.21	−2.46	(−∞, −2.46)	Level 0

If a student’s Rasch measurement of CT is below −2.46 logits, their CT is deemed at Level 0, signifying a lack of CT proficiency. At this stage, students have yet to demonstrate key CT skills and traits. They struggle to articulate the connections between two data sets and find it equally challenging to discern the underlying patterns or rules that govern data or the information structure. Students may also struggle to recognize patterns or similarities in problems, processes, solutions, or datasets.

The Rasch metric, ranging from −2.46 to −0.43, represents CT level 1, indicating that students have reached or surpassed the average measure for level 1 but have yet to reach the average measure for level 2 items. Performance indicators for this level include Ab2, Ab3, and Ge1. This level shows that students can clarify the relationships in complex datasets, discern the patterns or principles underlying the data and information structures, and identify recurring patterns or commonalities in problems, processes, solutions, or datasets.

A student’s CT abilities are categorized as Level 2 if the score lies between −0.43 and 0.67, highlighting performance markers such as Ab4, Al2, Al3, Ge2, and Ge3. At this level, students can construct precise models or simulations to describe the system’s operating mechanisms and future functions. Students can simultaneously perform multiple steps and devise the most efficient actions to solve a problem, eliminating redundant steps.

Level 3 requires a CT score between 0.67 and 2.4; at this level, the performance indicators are Al1, Al4, De1, De3, Ev1, Ev2, and Ev3. At this level, students construct a sequence of structured steps to address a problem and have the potential to execute the procedure automatically when faced with similar problems. They possess the ability to deconstruct problems and tasks, make complex issues more manageable, novel situations more comprehensible, and facilitate the design of large systems. Based on a comprehensive assessment, students determine the division of subtasks, evaluate resource utilization and solution applicability, and select the most effective and efficient method to solve the problem.

Finally, upon reaching a Rasch score of over 2.4 for CT, a student is classified under Level 4. The defining performance indicators for this level are Ab1 and De2. At this level, students are proficient in gathering essential and pertinent information from multiple sources and have the potential to analyze problems or tasks sequentially.

### 5.3 Differentiation of students’ CT

Students’ CT performance was categorized into various levels based on their Rasch scores (Figure 5). Most students (60%) who had participated in the study achieved Level 2 or Level 3 scores, whereas only 5% attained Level 4 scores. Almost a quarter of the participating students demonstrated CT proficiency of Level 1 or below, with 18% at Level 0 and 17% at Level 1.

**Figure 6:**
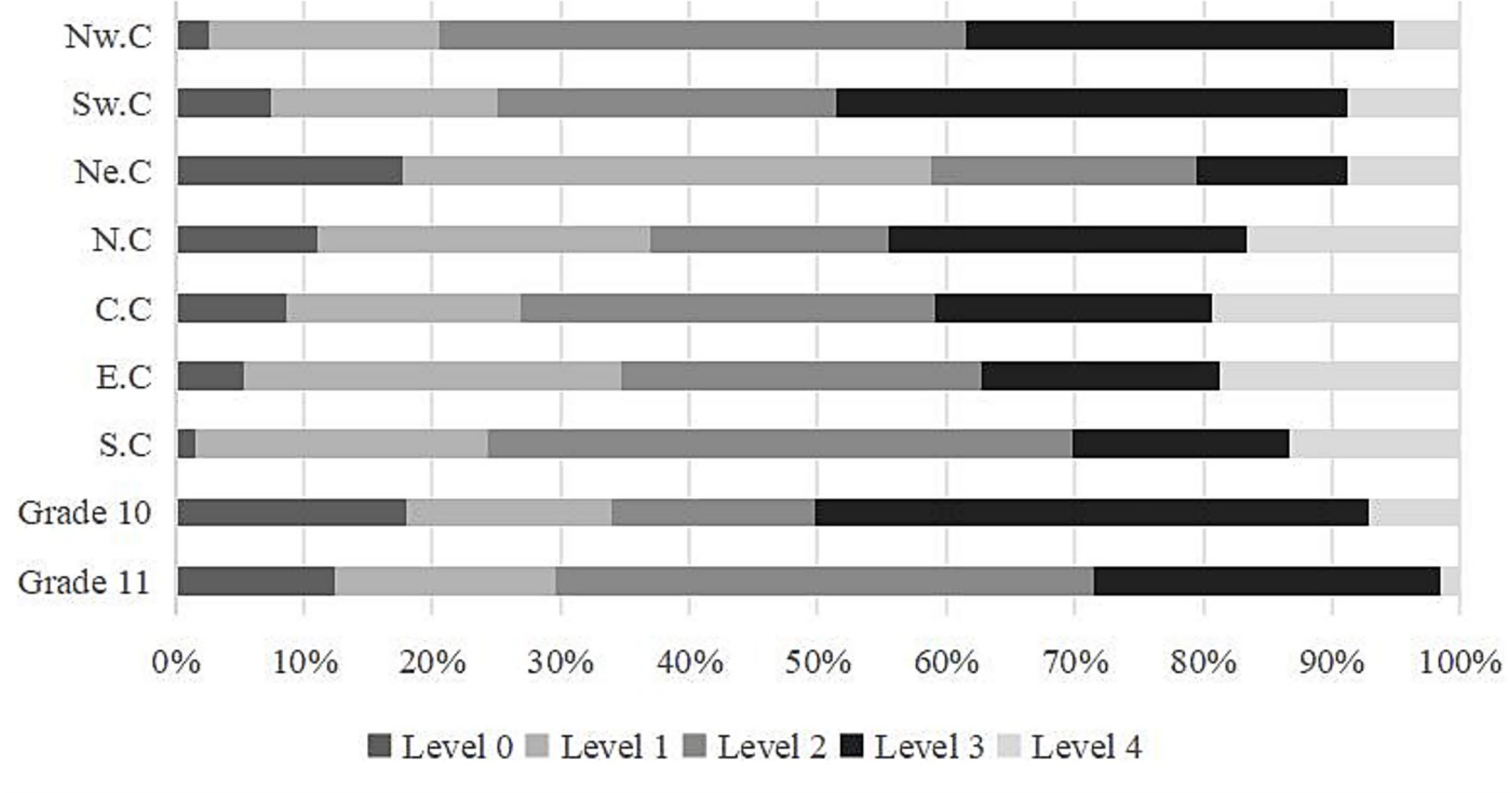
Differences in CT levels among students from different districts and grades. Note: NW C (Northwest China); SW C (Southwest China); NE C (Northeast China); N C (North China); C C (Central China); E C (East China); S C (South China).

To further verify whether the HS-CTOP indicator system and achievement level help students demonstrate outstanding advantages in CT, we compared the distribution of students’ CT levels across various districts and grades, as well as the score distribution across the 17 CT sub-dimensions. Figure 6 indicates that 60% students from Northeast China had a CT score of Level 1 or below, with 35–40% students from North and East China scoring at these levels. Students from Central, East, and North China had higher CT scores at Level 4 (15–20%). Students from Northwest China had the highest proportion at Level 3 (40%). Students from North China were polarized in their CT scores. However, the regional difference analysis of CT did not control for the influence of socio-economic background variables, these conclusions must be interpreted with caution.

Regarding grade differences and students’ CT levels, the overall CT scores of Grade 11 students were worse than those of Grade 10, with a greater number of students at Levels 0 and 1 in the former group. Further, the proportion of students in Grade 11 at Level 4 was lower than in Grade 10. Meanwhile, the CT performance of Grade 10 students was polarized, with 45% at Level 3. Among Grade 11 students, the same percentage was observed at Level 2, indicating a drop in their CT performance from Grade 10 to Grade 11. The lower CT proficiency among Grade 11 students may be attributed to the fact that Grade 11 biology content, such as “Homeostasis and Regulation”, focuses more on theoretical memorization than on experimental design, thereby reducing opportunities to practice CT. Alternatively, Grade 11 students may face greater pressure from college entrance exams, leading to less engagement in CT-related activities.

**Figure 7:**
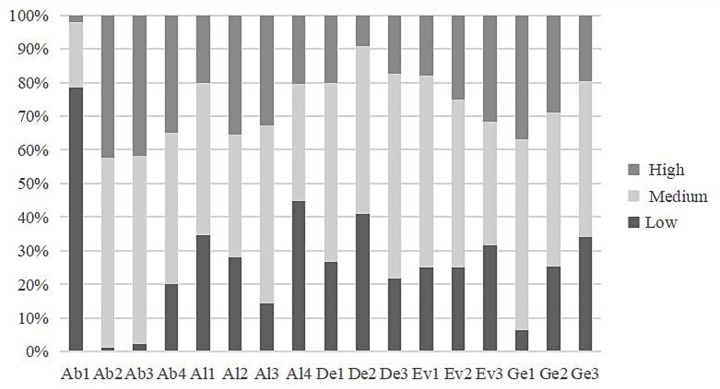
Distribution of CT scores according to sub-dimensions.

As mentioned earlier, each dimension of the HS-CTOP was scored using a partial credit Rasch model, with scores of “0, 1, 2” or “1, 2, 3” corresponding to low, medium, and high levels, respectively (Figure 7). Higher scores reflect better CT performance. Approximately 80% of the sampled students scored one on Ab1. Followed by six sub-dimensions, Al4, De2, Al1, Ge3, Al1, and Ev3, accounting for approximately 30–45%. The graph further illustrates that within the three sub-dimensions of Ab1, Al4, and De2, a minority of students were categorized into the high-performing group, with each segment comprising less than 10%. Moreover, students scored high on Ab2, Ab3, and Ge1.

## 6 Discussion and conclusion

### 6.1 HS-CTOP demonstrates strong reliability and validity

Although prior studies have developed multiple CT assessment tools (Çoban and Korkmaz, 2021; Luo et al., 2020; Tucker-Raymond et al., 2019), most of these instruments evaluated summative learning outcomes rather than monitoring the dynamic learning process. Limited studies have examined CT assessment in high school settings (Tang et al., 2020); moreover, context-specific assessment tools developed in research environments often lack ecological validity for implementation in real-world classrooms (Han, 2023). This study fills the gap in CT assessment by introducing an effective and reliable protocol, which is validated by Rasch model analysis, to monitor the CT development of high school students in science classrooms. The findings demonstrate that the HS-CTOP exhibits acceptable reliability and validity, which directly addresses and answers RQ 1 regarding the construct validity and internal reliability of the protocol and RQ 3 regarding the HS-CTOP’s discriminative ability across student grades and regions.

### 6.2 Validation of sub-dimensions and proficiency levels of CT

Existing CT assessment tools primarily focus on the ADEGA components (Tsai et al., 2021; Tsai et al., 2022) and offer limited refinement in CT assessments. This restricts the relevance of CT instructions and diminishes the impact of feedback on assessment outcomes on teaching methodologies. This study advances knowledge by intricately weaving CT into assessment methodologies, thereby creating precise and insightful indicators. Based on the Rasch model, we delineated 17 sub-dimensions and proficiency levels of students’ CT, findings that directly address RQ 2. These levels characterize students’ CT in science classrooms, providing educators and researchers actionable insights for curriculum design and skill enhancement.

### 6.3 Implications

#### 6.3.1 Theoretical and practical contributions to CT assessment

Notably, CT assessments that measure students’ learning should focus on assessing grades and emphasize the gaps in understanding (Grover, 2017). The HS-CTOP addresses this need by enabling dynamic tracking of CT proficiency at multiple levels and individual student analysis across 5 components and 17 sub-dimensions. However, notable disparities exist in performance across various aspects of CT, which aligns with the research of Korkmaz and Bai (2019). Specifically, the difficulty rating assigned to each item within the HS-CTOP reflects students’ effort to exhibit exemplary CT across various indicators. A higher difficulty rating, therefore, indicates that students’ CT performance failed to reach the desired standard for a specific indicator. To analyze the unsatisfactory performance on these indicators, we identified key factors, such as Ab1, Al4, and De2, which are critical for enhancing students’ CT proficiency. Item difficulty ratings within the HS-CTOP highlight critical gaps: students struggle with complex problem De and Ev, and demonstrate lower levels of difficulty with Ab and algorithmic thinking Al. This pattern underscores the need for targeted instruction in decomposition and evaluation skills, corroborating Selby (2015) research.

#### 6.3.2 Interpreting CT performance: the interplay of ability, instruction, and context

It is important to emphasize that the observed CT performance of students in this study is the result of the interplay between “students’ intrinsic abilities” and “instructional opportunities/task contexts,” rather than merely reflecting students’ inherent CT proficiency. From the perspective of instructional opportunities, the study found that Ab1 was the most challenging, likely not because of an inherently high cognitive demand in this subdimension, but because traditional high school biology classrooms often rely on predetermined data provided by teachers, leaving students with insufficient training opportunities for “autonomous selection of multi-source data”.

From the perspective of task contexts, the overall CT level of Grade 11 students was lower than that of Grade 10, primarily because Grade 11 biology content emphasizes theoretical memorization, with a reduced proportion of experimental design tasks, leading to fewer opportunities for CT practice rather than a decline in students’ CT. This finding suggests that the assessment results of the HS-CTOP can not only diagnose students’ CT levels but also reveal “CT cultivation gaps” in teaching. For example, if a class consistently scores low on Ab1, it may reflect a lack of training tasks involving multi-source data collection in instruction, rather than students inherently lacking this ability. Therefore, when using the HS-CTOP for teaching assessment, it is essential to interpret the results comprehensively in conjunction with specific instructional content and methods, avoiding the tendency to judge students’ CT abilities solely based on “scores.”

#### 6.3.3 Targeted implications for stakeholders

For research scholars, this protocol streamlines systematic assessment of performance differences across key geodemographic variables (e.g., district and grade level) and performance variations across various sub-dimensions. Pedagogically, it offers actionable insights for instructional leaders, enabling them to: (1) identify domain-specific competency gaps through detailed sub-dimensional analysis; (2) develop targeted interventions that align with proficiency benchmarks; (3) implement differentiated instructional scaffolding strategies. Notably, the protocol’s capacity to quantify regional performance disparities in policymakers’ empirical evidence will support the development of evidence-based policies to address inequities in CT education, particularly resource allocation and curriculum standardization.

#### 6.3.4 Boundaries of generalizability and interpretive caution

As an evidence-informed diagnostic instrument for high school science classrooms, the HS-CTOP enables targeted tracking of students’ CT development. However, its utility should be interpreted with caution given the absence of established criterion validity and validated solely in biology classrooms. The generalizability of the current findings is subject to several contextual constraints. First, the data were collected exclusively from 40-min high school biology lessons across seven regions of mainland China, limiting the protocol’s applicability to other science subjects or longer/shorter class durations. Second, only 6–8 students were randomly selected per class for observation, which may not fully represent the CT performance of the entire class. Third, observable CT behaviors varied across biology topics: for instance, CT sub-dimensions like De and Ab4 were more prominent in experimental modules, while Ge was less frequently observed in theoretical teaching modules (e.g., homeostasis). Future research should expand the sample to include diverse science subjects and teaching formats to enhance the generalizability of the HS-CTOP.

## 7 Limitations

Despite the distinct results, this study has several limitations. Initially, effective use of the protocol demands familiarity with core CT concepts and the dynamics of high school science classrooms, meaning novices require systematic training to ensure scoring consistency. Additionally, the protocol relies on high-quality video recordings and dedicated tools, which may be inaccessible in resource-limited contexts. To address these challenges, future applications could adopt optimized strategies: simplifying the coding process by selecting core sub-dimensions for routine assessments, developing standardized training materials to lower the learning curve, or integrating AI and other technologies to enhance efficiency while preserving the protocol’s process-oriented assessment advantage. These considerations offer practical guidance for real-world educational settings and highlight avenues for further tool refinement. Second, the analysis for RQ 3 (the discriminant validity of the HS-CTOP across different grades and regions) relied solely on descriptive statistics, future research should adopt inferential tests (e.g., chi-square tests, Kruskal-Wallis H tests) or Differential Item Functioning (DIF) analyses to rule out the interference of measurement bias on the research results. Third, a key methodological limitation lies in the nested data structure (students nested within classes and regions), which may lead to non-independent observations. Regional and grade-level comparisons could be affected by cluster effects. Future research should employ multilevel Rasch models to partition variance at the student, class, and regional levels, thereby isolating true ability differences from contextual factors. Fourth, owing to limitations in research design and external resources, we could not obtain criterion-related validity evidence for the HS-CTOP. Establishing the correlation between the scale’s results and those of related constructs, such as problem-solving ability, remains an area for future study.

## Data Availability

The original contributions presented in the study are included in the article/Supplementary material, further inquiries can be directed to the corresponding author.
