# Supplementary material for: Developing a process-oriented classroom observation protocol for assessing high school students’ computational thinking in science classrooms: a Rasch-based proficiency framework
Source: Front Psychol. 2026 Feb 20;17:1722422. doi: 10.3389/fpsyg.2026.1722422 (PMC12963286; doi:10.3389/fpsyg.2026.1722422)
Supplement: Supplementary file 1 [file Supplementary_file_1.docx]

***Supplementary Material:***

***Developing and validating an observation protocol for assessing high school students' computational thinking in science classrooms***

**1 Supplementary Material 1**

**Observation protocol for high school students' computational thinking in science classrooms (HS-CTOP) (Revised)**

| **Tier-1 indicators** | **Tier-2**  **indicators** | **Coding rubrics** |
| --- | --- | --- |
| Abstraction | (Ab1)  Data collection | 3: Students collect relevant/important information from multiple sources.  2: Students collect relevant information from two sources.  1: Students collect relevant information from only one source. |
|  | (Ab2)  Data analysis | 2: Students clarify the relationship between more than two datasets.  1: Students only clarify the relationship between two datasets.  0: Students fail to clarify the relationship between datasets. |
|  | (Ab3)  Pattern recognition | 2: Students identify the patterns/rules underlying the data/information structure.  1: Students identify part of the patterns/rules underlying the data/information structure.  0: Students fail to identify the patterns/rules underlying the data/information structure. |
|  | (Ab4) Modeling | 3: Students build correct models or simulations to indicate a system's future operations and functions.  2: Students build correct models or simulations to represent a system's operations.  1: Students build models or simulations, which are not entirely correct, to represent a system.  0: Students fail to build models or simulations to represent a system. |
| Algorithmic thinking | (Al1) Algorithm design | 2: Students create a sequential series of steps to solve a problem.  1: Students create a series of sequential steps to solve a problem, but not in order.  0: Students create incomplete steps to solve a problem. |
|  | (Al2) Parallelism | 2: Students implement more than two steps simultaneously.  1: Students conduct only two steps simultaneously.  0: Students implement only one step at a time. |
|  | (Al3) Efficiency | 2: Students design the fewest steps to generate potential solutions for a problem, eliminating redundant and unnecessary steps.  1: Students design fewer steps to solve a problem, but not least.  0: Students design several steps to solve a problem, but with several redundant and unnecessary parts. |
|  | (Al4) Automation | 2: Students execute the procedure automatically to solve similar problems.  1: Students execute the procedure when required to solve similar problems, but in a semi-automatic way.  0: Students execute the procedure when required to solve similar problems, but not in an automatic way. |
| Decomposition | (De1) Problem splitting | 2: Students break down complex problems/tasks to solve them easily as novel situations are better understood, or large systems are easier to design.  1: Students partially break down problems/tasks, not resulting in easier-to-manage parts.  0: Students are unable to break down problems/tasks. |
|  | (De2) Analytical thinking | 3: Students analyze and solve the given problems/tasks individually.  2: Students analyze and solve many parts of the given problems/tasks.  1: Students analyze but solve only some parts of the given problems/tasks. |
|  | (De3) Integration | 2: Students divide tasks into subtasks while integrating them to achieve the overall outcome.  1: Students divide tasks into subtasks without considering their integration.  0: Students determine dividing tasks into subtasks without considering their integration into a cohesive outcome. |
| Evaluation | (Ev1) Resource utilization | 2: Students based their judgments on the rationality, economy, and other aspects of resource utilization.  1: Students based their judgments on the rationality of resource utilization.  0: Students failed to make judgments regarding the use of resources. |
|  | (Ev2)  Target fitting | 3: Students accurately estimate if an algorithm/solution fits a given purpose.  2: Students estimate if an algorithm/solution fits a given purpose, but some inaccuracies exist.  1: Students partially estimate if an algorithm/solution fits a given purpose. |
|  | (Ev3)  Trade-off | 3: Students find the best (effective and efficient) solution for a problem.  2: Students evaluate the efficacy and efficiency of the solution to the problem.  1: Students only evaluate the efficacy of the solution to the problem. |
| Generalization | (Ge1) Identification | 2: Students identify patterns and commonalities in problems, processes, solutions, or data.  1: Students identify limited patterns and commonalities in problems, processes, solutions, or data.  0: Students fail to identify patterns or commonalities in problems, processes, solutions, or data. |
|  | (Ge2)  Adaptation | 2: Students adapt solutions to be applied to an entire class of similar problems.  1: Students adapt parts of solutions to an entire class of similar problems.  0: Students fail to adopt any solutions to similar problems. |
|  | (Ge3)  Transfer | 2: Students develop solutions that comprise modular, reusable components and leverage the modularity of their solution while working on the current problem and reusing pieces of previous solutions when confronting new challenges.  1: Students develop solutions that comprise modular and reusable components and leverage the modularity of their solution while working on the given problem.  0: Students develop solutions that comprise modular, reusable components but fail to transfer them to other contexts. |

**2 Supplementary Material 2**

**Coding example of "Al-Algorithmic thinking"**

| **Example** | **Analysis** |
| --- | --- |
| [T1] Our class had an inquiry group, and participants used the after-class time to perform pre-experiments. Now, let us invite a student from this group to share the experimental process and some of the problems encountered. Now, please welcome this student.  [S1] Our group's experiment aimed to explore the impact of different light intensity levels on the rate of photosynthesis. We believe that the rate of photosynthesis is high when light intensity is high. Our experimental independent variable is light intensity, with light bulbs of different wattage to control light intensity levels, because the variable is the rate of photosynthesis. We can measure the distance simultaneously to compare photosynthesis under different light intensities. This image shows our experimental device.  [S1] Our experimental device requires nine syringes, nine infusion tubes, one tube of red ink, three small bulbs of different wattage, and three cartons of the same size. Each carton is a black box: there is a hole for a minor bulb assembly. By cutting one side of the carton, we can place the syringe here to observe the experimental phenomenon and accurately observe and record the distance moved by the red ink. Our experiment aims to explore the effect of light intensity on the rate of photosynthesis; therefore, different cartons were installed with small bulbs of different wattage. Three groups of repeated experiments were performed under different light conditions to reduce experimental errors. Finally, we measured the average, which is the operational process of the entire experiment. Now, weigh nine groups of green chrysanthemums, each weighing 2 g. Put the Petri dish on the electronic scale, press the peel button to weigh the green chrysanthemum, and the line will be within the error range. | The teacher [T1] had asked students to design experiments to examine the impact of different light intensities on plant photosynthesis.  Students (e.g., [S1])designed and implemented a series of orderly steps to explore the premise of clarifying the independent, dependent, and irrelevant variables.  Therefore, the sub-dimension "algorithmic design (Al1)" under student [S1] algorithmic thinking (Al) is scored as 3.  In the experiment designed by student [S1], three repeated experiments were performed under each group of light intensity conditions, and the final average was measured to reduce the experimental error.  Further, the influence of multiple groups of light conditions on photosynthesis was explored.  Therefore, the sub-dimension "parallel (Al2)" under student [S1] algorithm thinking (Al) is scored as 2. |
| [S1] Now, assemble the experimental device, put the weighed green chrysanthemum into the syringe, and pour 50 ml of 2% sodium bicarbonate solution into the syringe. Now, O_2_ starts releasing. Once this is done, assemble nine sets of experimental devices.  [S1] As red ink was not used in the experiment, we chose Sudan Ⅲ as the droplet marker. Sudan Ⅲ dye was injected into the infusion tube with a syringe, connecting the infusion tube and syringe. Next, 5W, 9W, and 15W bulbs were placed in each carton, with three syringes under each carton. A piece of white paper was placed under the infusion tube to record the initial position of the Sudan Ⅲ red droplet every minute. We recorded 12 minutes of experimental data, as shown in the Figure.  [S1] This is the result of our experiment, and our group concluded that the greater the light intensity, the stronger the rate of photosynthesis. We encountered the following problems during the entire experiment. First, under the same light intensity, there is a difference in the distance traveled by the droplets in the three sets of infusion tubes, attributable to some syringes having more stems and others more leaves, leading to differences in the rate of photosynthesis in stems and leaves under the same light intensity. This may also be because the bulb is a point source of light that is direct and oblique, and has different effects, and the light intensity received in the green chrysanthemum is different. Second, the video shows the movement of droplets under a real light bulb in all three sets of experiments. After three minutes of the experiment, the speed of the middle droplet increased suddenly. Analysis suggests that the rapid dispersion of droplets is due to the sudden diffusion of the liquid. Therefore, why do droplets tend to disperse rather than gather? Sudan III sticks to the walls during movement, causing the droplets to disperse rapidly. Third, at the beginning of the experiment, there was no significant difference in the distance moved by the droplets under different light intensities. The chrysanthemum was placed in a dark environment from when the device was set up until the timing started. Subsequently, the chrysanthemum, a short-day plant, required time to adapt to the light environment.  [T1] Well, thank you, thank you very much! | The experimental process is concise, and each step is indispensable; it does not contain redundant steps.  Therefore, the sub-dimension "efficiency (Al3)" under student [S1] algorithm thinking (Al) is scored as 2.  During this process, there was no clear indication that student [S1] had solved similar problems.  Therefore, the sub-dimension "automation (Al4)" under student [S1] algorithm thinking (Al) is not assigned. |
